# A comprehensive review and update on acute severe lower gastrointestinal bleeding in Crohn’s disease: a management algorithm

**DOI:** 10.1093/gastro/goae099

**Published:** 2024-11-06

**Authors:** Tong Tu, Mengqi Chen, Zhirong Zeng, Jianming Lin, Luohai Chen, Caiguang Liu, Xiaojun Zhuang

**Affiliations:** Department of Gastroenterology, The First Affiliated Hospital, Sun Yat-sen University, Guangzhou, Guangdong, P. R. China; Department of Gastroenterology, The First Affiliated Hospital, Sun Yat-sen University, Guangzhou, Guangdong, P. R. China; Department of Gastroenterology, The First Affiliated Hospital, Sun Yat-sen University, Guangzhou, Guangdong, P. R. China; Department of Gastroenterology, The First Affiliated Hospital, Sun Yat-sen University, Guangzhou, Guangdong, P. R. China; Department of Gastroenterology, The First Affiliated Hospital, Sun Yat-sen University, Guangzhou, Guangdong, P. R. China; Department of Gastroenterology, The First Affiliated Hospital, Sun Yat-sen University, Guangzhou, Guangdong, P. R. China; Department of Gastroenterology, The First Affiliated Hospital, Sun Yat-sen University, Guangzhou, Guangdong, P. R. China

**Keywords:** Crohn’s Disease, gastrointestinal hemorrhage, evaluation, treatment, biological agent

## Abstract

Acute severe lower gastrointestinal bleeding is a rare but potentially fatal complication of Crohn's disease (CD), affecting between 0.6% and 5.5% of CD patients during their lifelong disease course. Managing bleeding episodes effectively hinges on vital resuscitation. Endoscopic evaluation and computed tomography play crucial roles in accurate identification and intervention. Fortunately, most bleeding episodes can be successfully managed through appropriate conservative treatment. Medical therapies, particularly infliximab, aim to induce and maintain mucosal healing and serve as the leading treatment approach. Minimally invasive procedures, such as endoscopic hemostasis and angio-embolization, can achieve immediate hemostasis. Surgical treatment is only considered a last resort when conservative therapies fail. Despite achieving hemostasis, the risk of rebleeding ranges from 19.0% to 50.5%. The objective of this review is to provide a comprehensive and updated overview of the clinical manifestations, diagnostic methods, therapeutic approaches, and prognostic outcomes associated with acute severe gastrointestinal bleeding in CD. Furthermore, we aimed to propose a management algorithm to assist clinicians in the effective management of this condition.

## Introduction

**Figure 1.**
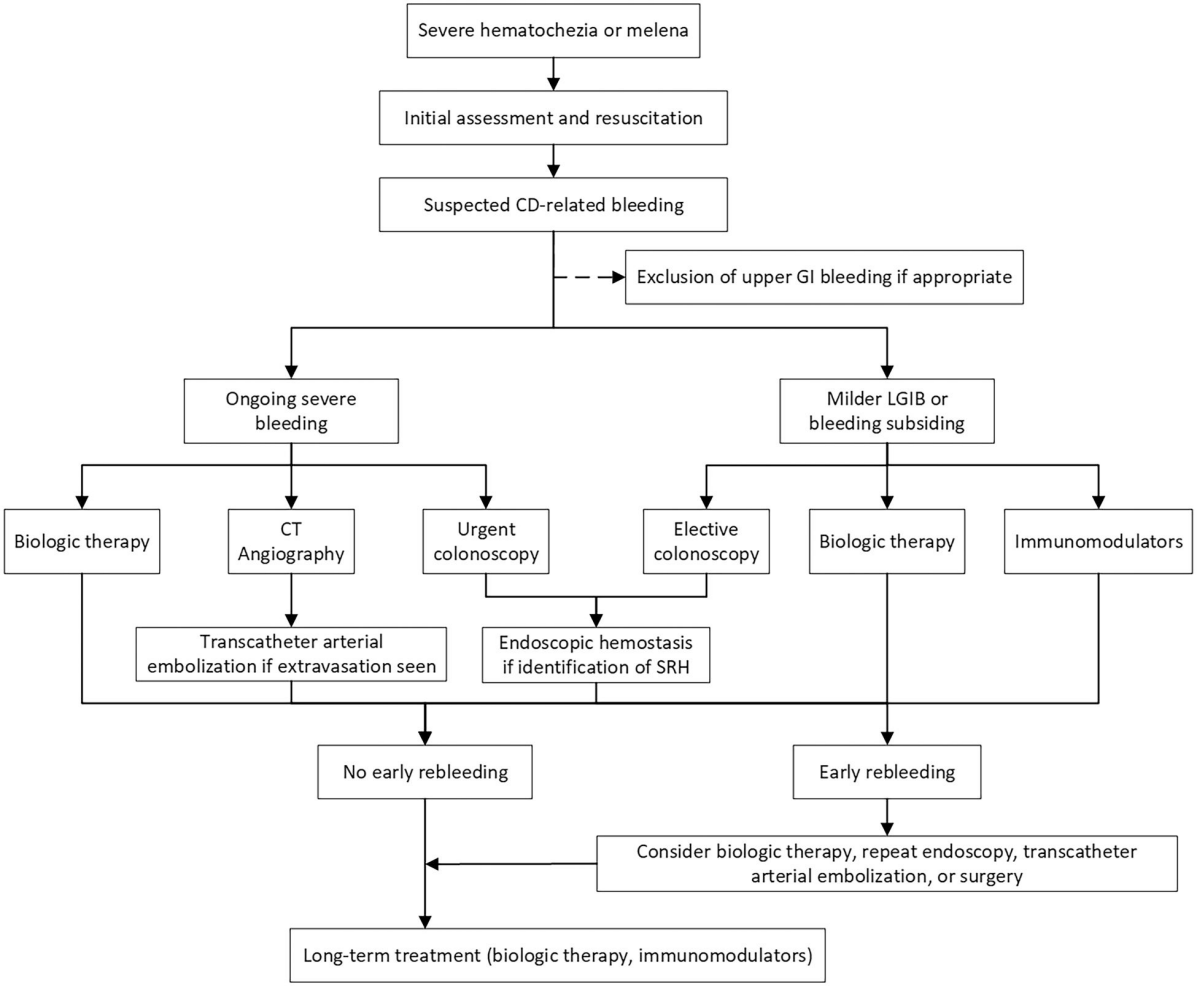
Management algorithm for acute severe lower gastrointestinal bleeding in Crohn’s disease. CD, Crohn’s disease; CT, computed tomography; GI, gastrointestinal; LGIB, lower gastrointestinal bleeding; SRH, stigmata of recent hemorrhage.

Acute severe lower gastrointestinal bleeding (LGIB) is one of the main acute complications in Crohn's disease (CD), firstly reported by Fallis *et al.* [1]. This manifestation is also known as the hemorrhagic form of CD [2], but limited evidence exists concerning this phenomenon, with only case reports and retrospective case series available. Although many randomized controlled trials addressing acute severe gastrointestinal bleeding excluded CD, their findings still have practical implications for the management of CD-related hemorrhage. Acute severe LGIB in CD presents significant challenges in diagnosis and treatment for the following reasons: (i) aggressive progression for the potentially life-threatening conditions; (ii) the difficulty in accurately localizing the bleeding source; and (iii) the high risk of rebleeding. Hence, this study aimed to provide a comprehensive overview of existing research on the characteristics, evaluation methods and treatment options, and propose a management algorithm for acute severe LGIB in CD (Figure 1).

## Characteristics

### Definition

Acute severe LGIB in CD is characterized by acute and massive hematochezia or melena, but the precise definition of acute severe LGIB in CD has evolved over time. Initially, studies focused on the requirement for blood transfusion to address hemodynamic instability resulting from acute severe LGIB [3–5]. Subsequent studies also considered the alteration in hemoglobin levels during bleeding episodes, such as a sudden drop below 9 g/dL or a decrease of at least 2 g/dL [2, 6, 7]. Most recently, Kim *et al.* [8] proposed a comprehensive definition encompassing profuse rectal bleeding leading to a sharp decrease in hemoglobin levels or the requirement for blood transfusion. They defined acute severe LGIB in CD as profuse rectal bleeding from the lower gastrointestinal tract resulting in (i) an abrupt decrease in hemoglobin level to <9 g/dL or at least 2 g/dL below the baseline, and/or (ii) the requirement of blood transfusion of at least two units within 24 h.

### Epidemiology

Acute severe LGIB occurs in approximately 0.6%–5.5% of CD patients during their clinical course [2–12]. Of these, 15.7%–34.7% experience it as the initial manifestation of CD [2, 8, 10]. Bleeding episodes may occur at any age or stage of CD, yet the average age is typically below 40 years, indicating the prevalence of CD among young individuals [2, 4, 8, 10, 12, 13]. In addition, CD also stands as the primary cause of small bowel bleeding in young individuals [14]. The time frame between CD diagnosis and the onset of bleeding shows considerable variation, ranging from 0 to 28 years.

### Location of bleeding

Precise localization of the bleeding site in hemorrhagic CD patients is crucial, given its potential occurrence anywhere along the gastrointestinal tract [15]. However, identifying the bleeding sites proves challenging due to the likelihood of multiple sites being involved and the spontaneous cessation of bleeding [6, 16]. Endoscopic evaluation should be conducted if the patient's condition permits, and additional imaging techniques like computed tomography angiography (CTA) or nuclear scintigraphy can assist in pinpointing the source of bleeding [15]. The average success rate for determining the bleeding location stands at 33.4% (ranging from 25.3% to 100%) [2, 4, 6, 8, 10, 17]. Belaiche *et al.* [2] noted that the bleeding sites were often located in the sigmoid or left colon (8/22), mainly due to the mechanical effect of formed stools in this area. However, Kim *et al.* [8] and Yoon *et al.* [10] reported that the small bowel emerged as the most common bleeding site among patients with identified bleeding sites (86.4% and 73.7%, respectively). Additionally, other bleeding sites such as ileocolic anastomosis and pseudopolyps of the ileum have also been observed [2].

### Inflammation burden

Only two retrospective studies have demonstrated the inflammation burden during episodes of acute severe LGIB in CD, but these studies did not find a prevalent high inflammatory burden in most patients with hemorrhagic CD. For example, Belaiche [2] *et al.* observed that 64.7% of patients (22/34) had quiescent CD at the time of bleeding. Similarly, Kim *et al.* [8] found that 77.8% of patients (14/18) had a Crohn's Disease Activity Index of less than 150, and 47.8% of patients (32/67) exhibited a normal range of C-reactive protein levels. However, Bauditz *et al.* [18] evaluated three patients with severe LGIB in CD who had a Crohn's Disease Activity Index score of 251 ± 31, which could been inflated due to anemia, along with a CD endoscopic index of severity of 13.1 ± 1.8.

### Potential pathogenesis

The pathogenesis of acute severe LGIB in CD patients remains uncertain. Many researchers speculated that transmural inflammation, particularly deep ulcers, could potentially erode large blood vessels, leading to massive gastrointestinal hemorrhage [2, 16, 19]. Alternatively, other researchers have proposed that major bleeding episodes may result from larger superficially located vessels with arteriovenous short circuits, as bleeding may not be associated with aggravated disease activity [18, 20]. The progression of CD to an advanced stage, characterized by reduced blood flow in the intestinal mucosa due to vascular thickening and scarring, may contribute to the prevention of bleeding events [21, 22].

### Risk factors for acute severe LGIB

The risk factors for hemorrhagic CD are heterogeneous because most evidence comes from retrospective investigations. Studies have revealed that the administration of azathioprine within 6 months of CD diagnosis or the utilization of azathioprine or 6-mercaptopurine before bleeding, could decrease the risk of hemorrhagic CD [8–10]. Furthermore, left colon involvement and a history of previous bleeding have been identified as risk factors for hemorrhagic CD, whereas the female gender has been linked to a decreased risk of such occurrences [9, 10].

## Diagnosis and evaluation

The diagnosis of CD involves a comprehensive assessment of clinical, radiographic, endoscopic, and histological characteristics. In young patients presenting with acute severe LGIB originating from unidentified intestinal ulcers, a prompt and accurate diagnosis is crucial to differentiate CD from other diseases such as ulcerative colitis, intestinal tuberculosis or intestinal Behcet's disease [4].

### Endoscopy

Given the infrequency of acute severe LGIB solely attributed to CD, hematochezia with hemodynamic instability may indicate an upper gastrointestinal bleeding event, prompting the necessity for an esophagogastroduodenoscopy examination to exclude such occurrences [23, 24]. For nearly all patients with acute severe LGIB, the primary investigative procedure is colonoscopy, enabling both the collection of biopsies for diagnostic purposes and the implementation of endoscopic hemostasis [23, 25]. The frequency of identifying bleeding sites through colonoscopy varies considerably, ranging from 10.6% to 60% [2, 8, 10]. Pardi *et al.* [6] also identified bleeding sites in two patients with CD (located in the duodenum and jejunum) by esophagogastroduodenoscopy. Conversely, Kim *et al.* [8] reported no positive findings via esophagogastroduodenoscopy in a cohort of 30 CD patients.

The optimal timing for conducting colonoscopy remains controversial. Early colonoscopy (24 h after the bleeding episode) has the potential to improve the identification of the bleeding source and the rate of endoscopic intervention [25, 26]. Nevertheless, early colonoscopy may not lead to meaningful clinical benefits such as reduced rebleeding incidents or mortality rates [26]. According to the current guideline from Europe, appropriate colonoscopy should only be performed after adequate bowel preparation [27]. A poorly cleaned colon could increase the risk of missing mucosal lesions and raise the chances of perforation compared with a well-cleaned colon [28]. Some studies still emphasized the presence of a large amount of blood in the intestine might act as a potent cathartic and bowel preparation does not increase the bleeding rate [29]. There is emerging evidence that urgent colonoscopy without bowel preparation may enhance the detection rate of the bleeding location in colonic diverticular bleeding, regardless of the presence of coagula and stool [30]. Moreover, hydroflush colonoscopy employing high-flow endoscopic irrigation and suction could serve as a substitute methodology even its accessibility remains limited [31]. Collectively, in cases of active severe bleeding, emergency colonoscopy without bowel preparation may be considered.

### Computed tomography

Computed tomography (CT) is essential for detecting bleeding sites and evaluating other complications, such as perforation, obstruction caused by stenosis, and abscesses. Guideline about small bowel bleeding recommended that CTA is preferred over CT enterography (CTE) in brisk active gastrointestinal bleeding [13]. One meta-analysis concluded that CTA has high sensitivity (85.2%) and high specificity (92.1%) in detecting active acute massive gastrointestinal bleeding [32]. However, the role of CT in patients with hemorrhagic CD is limited, with a diagnostic rate of 8.8%–19.6% for identifying bleeding sites [8, 10, 17, 33]. Enhanced CT identified bleeding sites through contrast extravasation in inflammation areas [17, 33]. However, in CD patients who typically have multiple wall abnormalities with hyperenhancement, bleeding sites may not be detectable unless there is a substantial amount of active bleeding [17]. Lee *et al.* [17] found that the diagnostic yield of CTE and non-enterographic CT did not obtain significantly different in acute severe LGIB of CD. Nagata *et al.* [34] found that the detection rate of vascular lesions was much higher in colonoscopy following CT than colonoscopy alone (35.7% vs 20.6%, *P *=* *0.01), leading to more endoscopic treatment.

### Nuclear scintigraphy

Although scintigraphy utilizing technetium-99-labeled red blood cells (RBC) can detect a bleeding threshold as low as 0.2–0.4 mL/min, it has limited effectiveness in hemorrhagic CD due to prolonged acquisition time, low diagnostic rate and inaccurate bleeding location [32, 35–37]. A retrospective study showed a similar incidence of active gastrointestinal bleeding of 38% between RBC scintigraphic scans and CTA, but the accurate incidence of bleeding site of CTA scans was significantly higher than that of RBC scintigraphic scans (53% vs 30%, *P *=* *0.008) [38]. Additionally, the presence of intestinal activity induced by hyperemia in CD can lead to an increased occurrence of false-positive outcomes in RBC scintigraphy [39].

## Treatment

### Resuscitation

The primary objective of initiating early resuscitative interventions is to sustain essential physiological parameters such as blood pressure, heart rate and blood oxygen to prevent organ damage [36]. In cases of hemodynamic instability, resuscitation is performed by intravenous administration of crystalloid fluids or blood products to normalize blood pressure and heart rate before endoscopic evaluation or intervention [24, 25, 40]. Packed RBC should be transfused to maintain hemoglobin levels above 7 g/dL. However, for patients encountering rapid bleeding alongside acute or chronic cardiovascular disease, a threshold of 8 g/dL should be considered [27, 40].

### Medical therapy

The purpose of medical therapy is to induce and maintain mucosal healing to achieve hemostasis without identifying bleeding locations. Medical therapy included mesalamine, 5-aminosalicylates, corticosteroids, immunosuppressant agents (azathioprine, 6-mercaptopurine and thalidomide), as well as anti-tumor necrosis factor (TNF) agents. Mesalamine and 5-aminosalicylates have exhibited restricted effectiveness in inducing and maintaining remission in CD and even do not manifest distinct advantages in the management of hemorrhagic CD [41]. In addition, the efficacy of corticosteroids in treating acute severe LGIB in CD remains a subject of ongoing debate. Belaiche *et al.* [2] found that 61.8% of patients (21 of 34) could successfully stop bleeding by corticosteroids. However, Aniwan *et al.* [42] revealed that all seven patients with hemorrhagic CD in their research continued to exhibit hematochezia despite being administered intravenous dexamethasone. Given its ineffectiveness in maintaining remission or healing mucosal lesions, its hemostatic effect is limited [43]. Immunosuppressant agents like thiopurine have exhibited superior efficacy compared with corticosteroids in achieving and maintaining mucosal healing, albeit with a delayed commencement of action spanning 12–17 weeks [44–46]. While thiopurine monotherapy may not be suitable for achieving rapid hemostasis, it can be valuable as a combination therapy with anti-TNF agents to enhance its effects [47, 48]. Additionally, thalidomide was noted to be effective in the cessation of LGIB in CD as early as 1997 [49], and it has also shown significant efficacy in managing LGIB related to intestinal angiodysplasia and refractory CD [50, 51]. For example, Bauditz *et al.* [18] reported successful cessation of severe refractory intestinal bleeding and steroid-resistant CD in three adults treated with a daily dose of 300 mg thalidomide. The bleeding stopped within two weeks after the start of thalidomide. After discontinuing, there is a possibility of recurring bleeding, but re-administration can likewise restore hemostasis. However, this high dose of thalidomide may lead to substantial side effects including transient fatigue and severe sedation [18]. Mechanistically, thalidomide exerts its effects by suppressing TNF for anti-inflammatory action, as well as downregulating vascular endothelial growth factor to deactivate the endothelium [52–54].

Infliximab has demonstrated remarkable efficacy in rapidly inducing and maintaining remission in patients with CD. Belaiche *et al.* [55] firstly reported the prompt cessation of bleeding with infliximab infusion (5 mg/kg) in patients with hemorrhagic CD in 2002. Subsequent case reports have consistently confirmed its effectiveness, even in patients who have failed corticosteroids and azathioprine [42, 56–59]. Almost all patients achieved bleeding cessation after 1–2 rounds of treatment with infliximab (5 mg/kg) [42, 59]. Until now, novel medical treatments including biological agents and small molecule drugs hold great promise in achieving rapid mucosal healing of CD as well [60–62]. However, their practical experience in the treatment of hemorrhagic CD remains limited and future studies will explore this possibility.

### Intervention therapy

One critical issue of undergoing an endoscopic examination is the identification of stigmata of recent hemorrhage, which encompass active bleeding, a non-bleeding vessel and an adherent clot [25, 36]. Stigmata of recent hemorrhage serves as a clear indication for endoscopic hemostasis procedures, such as hemoclips, laser or bipolar coagulation, as well as the injection of epinephrine or ethanolamine at sites of active bleeding [2, 6, 25, 63–65]. In a study by Belaiche *et al.* [2], 5 out of 7 patients with CD experiencing acute severe gastrointestinal bleeding successfully halted bleeding through endoscopic hemostasis. Similarly, Kim *et al.* [8] reported that three out of four patients with CD achieved hemostasis with endoscopic interventions. Recently, several innovative endoscopic hemostatic modalities have shown promising results in acute severe gastrointestinal bleeding unrelated CD. The utilization of over-the-scope clips in managing high-risk bleeding has exhibited a superior initial hemostatic effect and reduced risk of rebleeding [66]. This technology has proven effective in addressing bleeding associated with polypectomy or anastomotic ulcers, as well as bleeding from the anal transition zone [67]. Furthermore, topical hemostatic powders have shown effectiveness in treating lower LGIB [68].

Angiography and embolization should be considered for patients who do not respond adequately to hemodynamic resuscitation and may not tolerate bowel preparation and early colonoscopy [25]. Angiography has the capability to detect bleeding rates of 0.5–1 mL/min [23]. In hemorrhagic CD, the ileal branch emerged as the most frequently observed bleeding site during angiography [69]. The main advantage of angiography and embolization is the ability to halt severe gastrointestinal bleeding without the need for bowel preparation. CTA should be used as a diagnostic tool before angiography as it exhibits greater sensitivity than transcatheter angiography, detecting bleeding at a rate of 0.3 mL/min [36, 70]. The most common complication after embolization is the intestinal ischemia, which has reported the incidence of 1%–4% [24, 40]. In a retrospective study by Kim *et al.* [69], among 13 Crohn's disease patients with gastrointestinal bleeding who underwent transcatheter arterial embolization, 9 patients exhibited symptom improvement or achieved remission with conservative therapy within a 1-month follow-up period.

Arterial vasopressin therapy is also performed on patients who have positive findings through angiographs to stabilize their condition and postpone surgery [7, 21, 71, 72]. It is more effective for bleeding from hyperemic lesions [2, 21]. Vasopressin therapy involves initial infusion followed by repeated arteriography to assess bleeding cessation [72]. However, the prolonged duration of vasopressin infusion (12–24 h) and the requirement for a prolonged indwelling arterial catheter have led to a decreased preference for its use among doctors. Potential systemic side effects of intra-arterial vasopressin include hypertension, coronary vasoconstriction, arrhythmia, and intestinal ischemia [72–74].

### Surgery

Conservative treatment and endoscopy are generally sufficient to address acute severe LGIB related to CD [75, 76]. Surgery is reserved for patients with life-threatening bleeding, persistent hemodynamic instability, or those unresponsive to medical therapy or less invasive intervention [24]. In the era of biologics, the surgery rate for severe LGIB in hemorrhagic CD ranges from 4.3% to 7.6% [8, 10, 12]. Yao *et al.* conducted a retrospective study showing that acute severe LGIB accounted for 10.8% (9 of 83) of complications leading to surgery within one year of a new CD diagnosis [77]. Prior to surgical resection, pinpointing the bleeding site is crucial to prevent post-surgery rebleeding from the unresected source and to mitigate excess mortality following ‘blind’ total colectomy [24, 25, 40]. Abdominal enhanced CT stands out as the preferred diagnostic tool, capable of identifying complications like bleeding, perforation, stenosis-induced obstruction, and abscess presence [40]. In unstable patients with hemorrhagic shock undergoing urgent surgery, intra-operative endoscopy, if available, could be useful in localizing the bleeding source [25].

### Long-term treatment

In hemorrhagic CD patients who achieve hemostasis, particularly when bleeding spontaneously ceases, immunomodulators or biologic therapy should be employed to achieve mucosal healing. Biologics are the preferred treatment option in all instances, and patients who successfully control bleeding with biologics should continue the same treatment for long-term maintenance. Although both thalidomide and azathioprine can gradually achieve mucosal healing, thalidomide is superior to azathioprine due to its efficacy in managing recurrent bleeding [51, 78, 79].

## Prognosis

### Mortality

The mortality rate of acute severe LGIB in CD ranges from 0% to 14.3% [4, 5, 9, 55]. Direct deaths resulting from episodes of acute severe LGIB were relatively rare [9]. The majority of the death cases were associated with systemic dysfunction or comorbidities such as sepsis, hematological diseases, pneumonia, acute ventricular fibrillation, and so on [4, 5, 9].

### Rebleeding

The rebleeding rate of acute severe LGIB has been reported from 19.0% to 50.5% [4, 6, 8, 9, 12]. The studies focused on rebleeding episodes of acute severe LGIB remain limited. Li *et al.* identified age ≥36 years old and bleeding episodes >3 months before admission as positive predictors for recurrent episodes [9]. Lee *et al*. revealed that the extensive CD and bowel wall-to-artery enhancement ratio assessed by CTE indicated a high risk of rebleeding [17]. Regarding treatment, Li *et al.* revealed that surgery was independently associated with a reduced risk of rebleeding [9]. However, Kim *et al.* did not draw the same conclusion about the effectiveness of surgery due to the low surgery rate [12]. They further revealed that anti-TNF therapy could reduce the risk of rebleeding in anti-TNF-naïve patients.

### Effect on disease course

Acute severe LGIB in CD does not significantly impact the long-term course of the disease [5, 10]. Yoon *et al.* conducted a retrospective matched cohort analysis and found no significant differences in the cumulative risks of behavioral progression and intestinal resection between the bleeding and non-bleeding groups after the bleeding/index date [10]. The hospitalization rate was higher in the bleeding group compared with the matched non-bleeding group. However, when hospitalizations due to rebleeding episodes were excluded from the analysis, the hospitalization rate was not significantly different between the bleeding and matched non-bleeding groups.

## Conclusions

Although mild LGIB is common in patients with CD, acute severe LGIB is a rare but critical condition that necessitates prompt diagnosis and intervention. We proposed a management algorithm of acute severe LGIB in CD to assist clinicians to manage this condition. The initial management of hemorrhagic CD focuses on stabilizing hemodynamics. CTA is helpful for rapid diagnosis and assessment. Colonoscopy serves both diagnostic and therapeutic purposes and can be scheduled electively. Infliximab has shown significant efficacy in achieving rapid hemostasis and preventing rebleeding. Although the direct mortality rate associated with acute severe LGIB in CD is low, the risk of rebleeding remains significant. Therefore, additional research is necessary to better understand the underlying mechanisms of this disease. Future studies should concentrate on elucidating the pathophysiological pathways involved in acute severe LGIB in CD and assessing the efficacy and safety profiles of various treatment modalities.

## Authors’ contributions

Z.Z. and X.Z. conceived the study and designed the research. T.T., J.L., C.L., and M.C. did the literature search. T.T. drafted the manuscript. All authors critically revised the manuscript and approved the final version of the manuscript.
